# Editorial: Invertebrate Neurobiology: Sensory Systems, Information Integration, Locomotor- and Behavioral Output

**DOI:** 10.3389/fphys.2021.807521

**Published:** 2021-11-26

**Authors:** Philippe Lucas, Sylvia Anton

**Affiliations:** ^1^INRAE, Sorbonne Université, INRAE, CNRS, UPEC, IRD, Université de Paris, Institute of Ecology and Environmental Sciences of Paris, Paris, France; ^2^INRAE/Agrocampus Ouest/Université Rennes 1, Institute of Genetics, Environment and Plant Protection, Agrocampus Ouest, Angers, France

**Keywords:** invertebrate, neurophysiology, neuroanatomy, locomotion, sensory cues

Invertebrates are a very diverse group of animals, which have developed sophisticated sensory systems, brain functions and motor systems to respond with adaptive behavior to signals emanating from various environments of biotic and abiotic origin. Strong sensory and cognitive capacities are found in invertebrates with small brains, which can therefore serve as model organisms to understand nervous system function also in organisms with bigger brains (Chittka and Niven, 2009). As a follow-up of a conference on the neurobiology of invertebrates in Versailles/France in May 2018, we collected articles in the present Research Topic in Frontiers in Invertebrate Physiology on various aspects of invertebrate neurobiology in different taxa, reaching from molecular over cellular studies up to sensory, brain and motor function and its plasticity, as well as behavioral output. We also include recent methodological developments, improving the feasibility of studies in these fields. The Research Topic comprises 17 original Research articles and 5 Reviews.

A first group of articles treats various aspects of insect olfaction. Identification, expression and functional analyses of antennal olfactory genes are presented in the rice grasshopper, two moth species, and a migratory locust. In the rice grasshopper, *Oxya chinensis*, different olfactory genes coding for odorant-binding proteins, chemosensory proteins, sensory neuron membrane proteins, as well as odorant and ionotropic receptors have been identified. For some of these genes, sex-biased expression was found (Cui et al.). An odorant receptor (OR) for repellents in the Asian cornborer *Ostrinia furnacalis*, and a plant odor-specific OR in the oriental armyworm *Mythimna separata* were functionally characterized using heterologous expression in *Xenopus* oocytes (Yu et al.; Zhang et al.). In the locust *Schistocerca gregaria*, Jiang et al. mapped the presence of a large number of ORs on the antennal sensillum types and found primarily a single OR expressed in each sensillum. However, some OR-specific cell clusters in certain sensilla were formed during development, as shown by comparing the expression of ORs between different nymph stages. Two reviews concern the characteristics of odorant stimuli, crucial for electrophysiological and behavioral experiments, taking the ecology of insects into consideration. A first review article treats the characterization of dynamic odorant stimuli (Pannunzi and Nowotny), summarizing the critical questions to define odor concentrations in space and their dynamics, as well as the spatio-temporal structure of odor stimuli in a natural environment from an experimental and theoretical point of view. Conchou et al. review what is known on insect odorscapes, i.e., the complex odor distribution in a natural environment, how insects deal with it and how odorscapes might be manipulated for sustainable pest control. Two electrophysiological studies investigate odor responses at different levels of the olfactory pathway. Coding of slowly fluctuating olfactory cues is described in so-called ON-OFF olfactory receptor neurons in the cockroach *Periplaneta americana* (Hellwig et al.) and modulation of odor responses within the lateral horn, a superior olfactory brain center was shown to enhance bilateral contrast of odor inputs in the fruitfly *Drosophila melanogaster* (Mohamed et al.). Another innovative study describes the peripheral and central olfactory system and odor-guided behavior in head lice *Pediculus humanis capitis*, highly specialized insects which have hardly been studied before (Ortega Insaurralde et al.).

A second series of articles deals with plasticity of chemosensory systems and methodological considerations to study this. Hostachy, Couzi, Hanafi-Portier, et al. describe experimental parameters to be taken into account when testing proboscis extension responses to sugar solutions in the moth *Agrotis ipsilon*. The same authors subsequently test the influence of pre-exposure with conspecific and heterospecific sex pheromones on gustatory habituation and find effects after different delays depending on the pheromone used, thus suggesting that different central pathways are implicated in the different observed effects (Hostachy, Couzi, Portemer et al.). Nouvian and Galizia describe a newly developed device for aversive learning in freely moving honey bees: an automated Y-maze, suitable to test olfactory or visual cues. A study in the fruitfly *Drosophila melanogaster* demonstrates elegantly that thermogenetically generated stochastic activity patterns of olfactory projection neurons can replace conditioned stimuli within mushroom body neurons (Warth Pérez Arias et al.). In the cockroach *Periplaneta americana*, both classical and operant learning paradigms using olfactory cues and a sucrose reward revealed inter-individual learning differences, comparable to what has been found earlier in honey bees and humans (Arican et al.). Extending on the topic of individuality in insects, Sánchez-Alcañiz and Molla Albaladejo review the genetic bases of behavioral individuality in *Drosophila melanogaster*, which occurs in olfactory-guided behavior and olfactory learning, but also in various other contexts.

A different sensory modality is investigated in another paper. Meiser et al. studied modulation of the sensitivity of touch cells in the leech. They found a non-synaptic mechanism switching the response behavior of these sensory neurons from rapidly to slowly adapting spiking.

Four other papers deal with locomotor control and its modulation in different invertebrates. Emanuel et al. review what is known on the role of different regions in the central nervous system in controlling posture and locomotion in insects. Two original articles deal with the role of serotonin (5-HT) on influencing motoneurons and behavioral output. In crayfish, a single 5-HT neuron was shown to differentially modulate motoneurons (simultaneous excitatory and inhibitory effects in different leg motoneurons) in the abdominal ganglia (Bacqué-Cazenave et al.). In the pond snail *Lymnaea stagnalis*, serotonin seems to influence accelerated locomotion, which subsequently improves cognitive abilities (decision-making under uncertainty) (Aonuma et al.). Another review article presents the current knowledge on a specific group of clock neurons in *Drosophila melanogaster*, DN1p neurons, and specifically their role in the circadian regulation of motor control. Lamaze and Stanewsky specifically discuss that these neurons might be responsible for two locomotion peaks per day in constant darkness.

Another paper deals with a nicotinic acetylcholine receptor subunit in cockroaches. These receptors are important for synaptic transmission in the brain and represent targets for neonicotinoid insecticides. Here a new receptor subunit was characterized and its expression in the brain described (Cartereau et al.).

Finally, a research paper demonstrates how wing scale pigmentation in the Bogong moth, *Agrotis infusa*, leads to visual camouflage within its natural environment (Stavenga et al.).

Taken together, this Research Topic expands our view on recent advances in various domains within invertebrate neurobiology by assembling original articles using multidisciplinary approaches and reviews on major themes of interest.

## Author Contributions

Both authors listed have made a substantial, direct, and intellectual contribution to the work and approved it for publication.

## Conflict of Interest

The authors declare that the research was conducted in the absence of any commercial or financial relationships that could be construed as a potential conflict of interest.

## Publisher's Note

All claims expressed in this article are solely those of the authors and do not necessarily represent those of their affiliated organizations, or those of the publisher, the editors and the reviewers. Any product that may be evaluated in this article, or claim that may be made by its manufacturer, is not guaranteed or endorsed by the publisher.
